# SARS-CoV-2 NSP14 MTase activity is critical for inducing canonical NF-κB activation

**DOI:** 10.1042/BSR20231418

**Published:** 2024-01-09

**Authors:** Marie J. Tofaute, Benjamin Weller, Carina Graß, Hridi Halder, Bushra Dohai, Pascal Falter-Braun, Daniel Krappmann

**Affiliations:** 1 Research Unit Signaling and Translation, Group Signaling and Immunity, Molecular Targets and Therapeutics Center, Helmholtz Zentrum München – German Research Center for Environmental Health, Neuherberg, Germany; 2 Institute of Network Biology (INET), Molecular Targets and Therapeutics Center (MTTC), Helmholtz Zentrum München, German Research Center for Environmental Health, Munich-Neuherberg, Germany; 3 Microbe-Host Interactions, Faculty of Biology, Ludwig-Maximilians-Universität (LMU) München, Planegg-Martinsried, Germany

**Keywords:** host-pathogen interactions, methyltransferases, nuclear factor kappaB

## Abstract

Upon SARS-CoV-2 infection, patients with severe forms of COVID-19 often suffer from a dysregulated immune response and hyperinflammation. Aberrant expression of cytokines and chemokines is associated with strong activation of the immunoregulatory transcription factor NF-κB, which can be directly induced by the SARS-CoV-2 protein NSP14. Here, we use NSP14 mutants and generated cells with host factor knockouts (KOs) in the NF-κB signaling pathways to characterize the molecular mechanism of NSP14-induced NF-κB activation. We demonstrate that full-length NSP14 requires methyltransferase (MTase) activity to drive NF-κB induction. NSP14 WT, but not an MTase-defective mutant, is poorly expressed and inherent post-translational instability is mediated by proteasomal degradation. Binding of SARS-CoV-2 NSP10 or addition of the co-factor S-adenosylmethionine (SAM) stabilizes NSP14 and augments its potential to activate NF-κB. Using CRISPR/Cas9-engineered KO cells, we demonstrate that NSP14 stimulation of canonical NF-κB activation relies on NF-κB factor p65/RELA downstream of the NEMO/IKK complex, while c-Rel or non-canonical RelB are not required to induce NF-κB transcriptional activity. However, NSP14 overexpression is unable to induce canonical IκB kinase β (IKKβ)/NF-κB signaling and in co-immunoprecipitation assays we do not detect stable associations between NSP14 and NEMO or p65, suggesting that NSP14 activates NF-κB indirectly through its methyltransferase activity. Taken together, our data provide a framework how NSP14 can augment basal NF-κB activation, which may enhance cytokine expression in SARS-CoV-2 infected cells.

## Introduction

Following its emergence in the city of Wuhan, China, in December 2019 [1], the SARS-CoV-2 virus has caused a global pandemic with more than 700 million cases and 6.8 million deaths worldwide (estimated in April 2023) (WHO, 2023). Symptoms in hospitalized COVID-19 patients range from fever, dry cough and shortness of breath to complications such as pneumonia, acute respiratory distress syndrome and acute respiratory failure [2]. In the 5% of COVID-19 patients who require intensive care [2], features of a cytokine storm syndrome are observed, manifesting as dysregulated systemic hyperinflammation [3–5]. Ultimately, this hypercytokinemia is associated with multiorgan failure and is a driving cause of fatal outcome [4,5]. The degree of hypercytokinemia also directly correlates with disease severity [6] and has even been suggested as a prognostic factor for severe disease progression [3,7]. Despite the rapid development of several vaccines [8,9], new SARS-CoV-2 variants and novel coronaviruses are expected to emerge in the near future, requiring treatment options for severe cases.

SARS-CoV-2 is an enveloped single-stranded RNA virus with a large genome of approximately 30 kb consisting of 14 open reading frames (ORFs) [10]. Two-thirds of the genome consists of overlapping ORF1a and ORF1b, which encode the polyproteins pp1a and pp1b, containing the non-structural proteins (NSPs) 1-16 [10]. The remainder of the genome encodes the four structural proteins, which are the building blocks of new virions, and eight accessory proteins, which contribute to viral survival and replication [10].

After viral protease processing, the viral replication-transcription complex (RTC) assembles with the enzymatically active NSP 12-16 and the accessory proteins NSP2-11 [10]. In this RTC, NSP14 as a bifunctional protein contains a N-terminal exonuclease (ExoN) domain, a linker region and a C-terminal N7-methyltransferase (MTase) domain [11]. The structure and the enzymatic domains of SARS-CoV-2 NSP14 are highly homologous to NSP14 from other coronaviruses that infect humans, which is why many findings from previous studies with SARS-CoV-1 and MERS-CoV also apply to SARS-CoV-2 proteins. The exonuclease activity ensures genomic stability by proofreading and excising incorrectly inserted nucleotides during replication [12,13] and is essential for SARS-CoV-2 replication [14]. The ExoN domain is related to the DEDD exonuclease superfamily [15] and its catalytic center consists of four conserved acidic residues, D90, E92, E191 and D273 [11,15]. Upon binding of its cofactor NSP10 with its N-terminal domain, NSP14 undergoes substantial conformational changes that enable catalytic activity [11,12,16] and significantly increase the stability of the NSP14 protein [17]. The N7-MTase domain catalyzes the methylation of the cap guanine at position N7, which is an intermediate step in the viral RNA capping process [18]. Viral RNA capping contributes to survival and replication by protecting the viral RNA from host cell exonucleases, circumventing antiviral responses and allowing translation by the host machinery [13]. The active site binds the methyl donor S-adenosylmethionine (SAM) at amino acids (aa)331-338 [19] and brings it into close proximity to the RNA substrate to facilitate methyl transfer [11]. Interestingly, N-terminal deletions also impair methyl transfer activity, suggesting some cooperative structural effects [19].

A widely used approach to understand how SARS-CoV-2 disrupts the human cell homeostasis is the systematic mapping of viral-human protein–protein interactions [20–25]. In a large-scale interactome mapping effort, we discovered that NSP14 is able to interact with several NF-κB pathway proteins, specifically TRAF2 (TNF receptor associated factor 2), NEMO (NF-κB essential modulator) and cRel [26]). Indeed, we could demonstrate the induction of transcriptional NF-κB activity in a dose dependent manner by overexpressing NSP14. This finding is consistent with other publications establishing NSP14 as a viral factor that activates NF-κB [27,28].

Gradual upregulation of the NF-κB pathway with disease exacerbation is revealed also in COVID-19 patients, by correlating disease severity grades with up-regulated genes in blood [6] and bronchoalveolar lavage fluid samples [29] as well as with proteomic changes in patient lung tissue [30]. Further, *in vitro* experiments highlight the up-regulation of TNFα signaling via activation of NF-κB as an initial response starting 6 h after infection [31]. Thus, activation of NF-κB, which is linked to the induction of immune responses and inflammation [32], is also central to cytokine release after SARS-Cov-2 infection and may thus contribute to COVID-19 severity.

To date, three independent studies have demonstrated that NSP14 can directly induce NF-κB activation in host cells [26–28]. However, details on the molecular mechanism of NSP14-triggered NF-κB activation and the requirements of cellular host factors are largely lacking. By dissecting the role of each NSP14 domain and its enzymatic functions, we not only demonstrated the essentiality of methyltransferase activity for NF-κB activation but also discovered its importance for NSP14 protein stability. In addition, we identified the NF-κB subunit p65 as the main driver for NSP14-induced NF-κB transcriptional activity. Further, we provide evidence that NSP14 alone is unable to activate the canonical NF-κB signaling pathway or to induce a full response of endogenous NF-κB target genes. Instead, it mainly augments basal NF-κB activity.

## Results

### Functional NSP14 N7-MTase domain is essential for activating NF-κB

**Figure 1:**
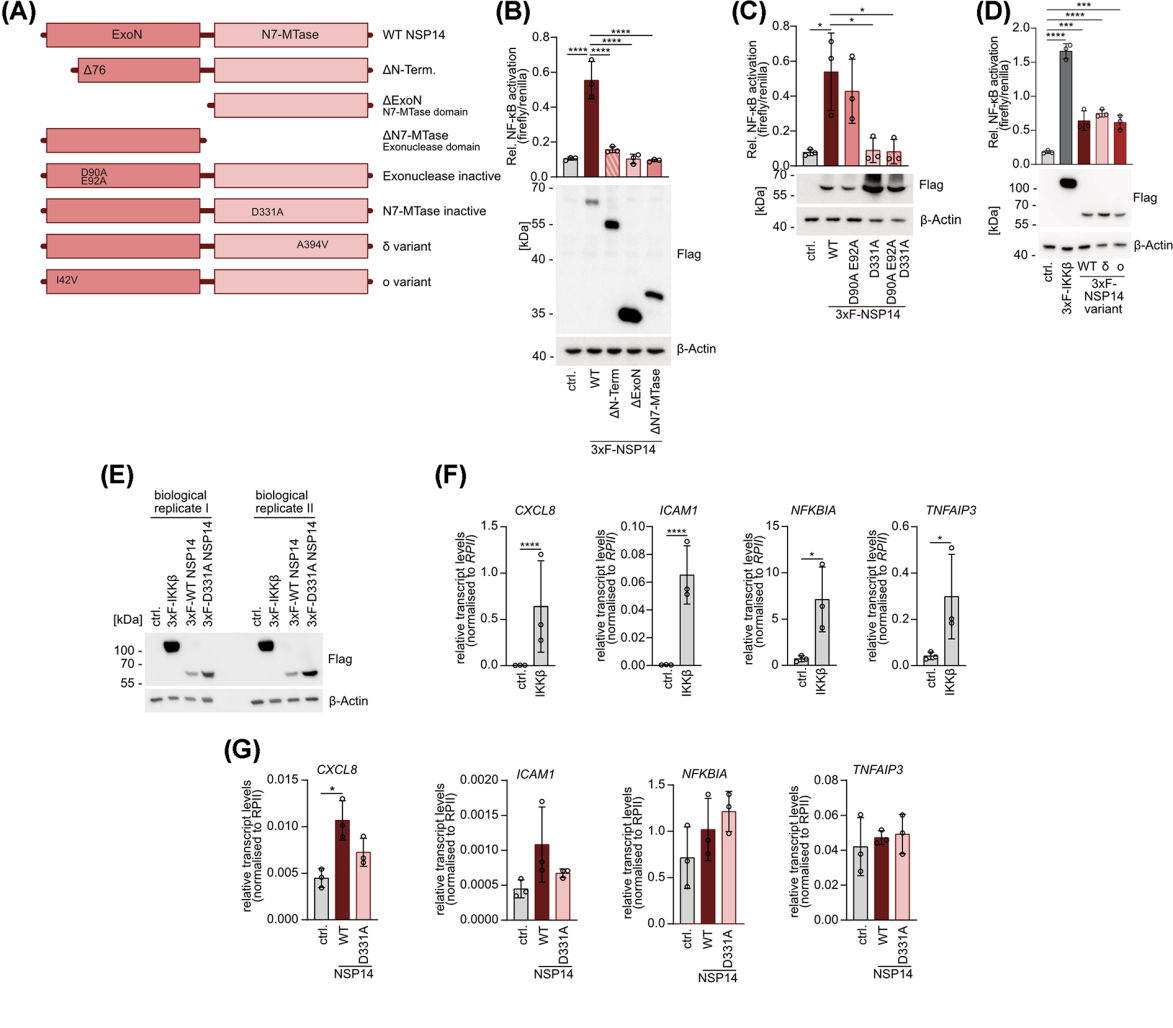
Full length NSP14 with methyltransferase activity induces NF-κB activity (**A**) Domain structure of WT NSP14 and generated truncation and point mutants analyzed in this study. (**B-D**) NF-κB reporter assays in HEK293 cells upon overexpression of (B) NSP14 truncation mutants, (C) catalytically inactive NSP14 point mutants and (D) NSP14 mutants from SARS-CoV-2 variants with representative Western blot for determination of protein expression levels. Relative NF-κB activation is determined by normalizing NF-κB-firefly luminescence to Tk-renilla luminescence (mean ± SD, *n*=3). (**E**) Expression levels of transfected NSP14 and IKKβ in two representative biological replicates analyzed in Western blot. (**F**) Expression of NF-κB target genes *CXCL8*, *ICAM1*, *NFKBIA* and *TNFAIP3* in HEK293 cells overexpressing IKKβ, quantified in RT-PCR. Values measured for target genes were normalized to RPII transcript levels (mean ± SD, *n*=3). (**G**) Expression of NF-κB target genes *CXCL8*, *ICAM1*, *NFKBIA* and *TNFAIP3* in HEK293 cells overexpressing NSP14, quantified in RT-PCR. Values measured for target genes were normalized to RPII transcript levels (mean ± SD, *n*=3). Significances were compared by unpaired Student’s *t*-test (**F**) or one-way analysis of variance (ANOVA) combined with (B,C,G) Tukey’s or (D) Dunnett’s multiple comparison test; *P≤0.05, ***P≤0.001, ****P≤0.0001.

To elucidate how NSP14 mechanistically affects host cell NF-κB signaling, we generated several NSP14 deletion or missense mutants (Figure 1A). NSP14 WT and mutants were overexpressed in HEK293 cells, activation of NF-κB was measured by dual luciferase-NF-κB reporter assay and expression was determined by Western blotting (Figure 1B-D). Despite higher protein levels compared to NSP14 WT, truncation of either the entire exonuclease (ΔExoN) or the methyl transferase (ΔN7-MTase) domain abolished NF-κB activation (Figure 1B). Further, deletion of the N-terminus, which serves as the binding interface for the cofactor NSP10 and is needed for effective N7-MTase activity, also abrogated NF-κB induction. Next, we focused on the role of NSP14 enzymatic activity and genetically inactivated the two active centers in the ExoN (D90A E92A) and the N7-Mtase domain (D331A) in combination or alone. While loss of ExoN activity did not affect NF-κB activation, catalytic inactivation of the N7-MTase in NSP14 completely prevented the NF-κB activating function either alone or in combination with the ExoN mutations (Figure 1C).

The NSP14 construct used to study effects on NF-κB activation corresponds to the original SARS-CoV-2 strain (Wuhan-Hu-1; NCBI reference NC_045512.2). As a variety of SARS-CoV-2 variants emerged during the pandemic, we were interested in whether natural amino acid exchanges that have been found in NSP14 alter its potential in NF-κB activation. We generated the δ-derived variant of NSP14 by introducing an exchange in the MTase domain (A394V) and the ο-derived variant that contains a variation of the ExoN domain (I42V). All natural NSP14 variants showed an equivalent expression on protein level and activated NF-κB to a similar extend (Figure 1D). Thus, full length NSP14 of different SARS-CoV-2 strains are able to induce NF-κB, which relies on functional N7-MTase activity.

NSP14 is able to induce NF-κB reporter gene activation and we wanted to determine whether NSP14 alone is also able to induce expression of endogenous NF-κB target genes, as it has been suggested by transcriptomic analyses [28]. Thus, we measured induction of selected well-characterized NF-κB target genes (*CXCL8*, *ICAM1*, *NFKBIA* and *TNFAIP3*) after expression of NSP14 or IKKβ in HEK293 cells (Figure 1E-G). While IKKβ was strongly increasing transcript levels of all four genes (Figure 1F), there was only a very mild significant increase of *CXCL8* transcripts detected upon expression of WT NSP14 but not the methyltransferase-defective D331A mutant (Figure 1G). In addition, there was a tendency for enhanced *ICAM1* mRNA transcription, but all other target genes were not induced in HEK293 cells. Taken together, this suggests that in contrast with IKKβ, NSP14 expression alone is unable to induce a full transcriptional response of endogenous NF-κB target genes.

### NSP10 and SAM stabilize NSP14 protein

**Figure 2:**
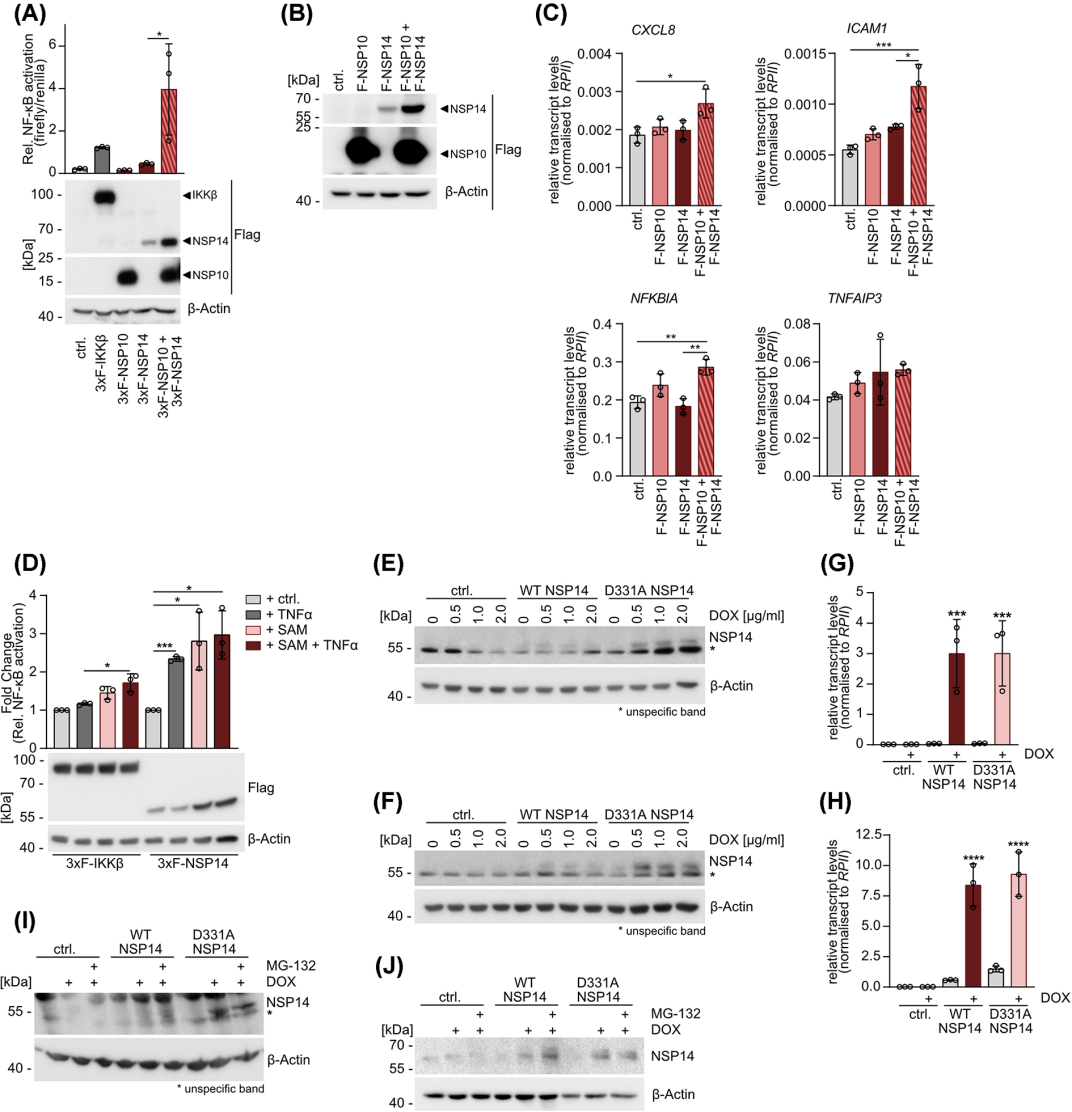
NSP14 protein instability can be counterbalanced by cofactors of proteasomal inhibition (**A**) NF-κB reporter assay in HEK293 cells overexpressing NSP14, NSP10 or both and representative quantification of protein expression levels by Western blot. (**B**) Expression levels of single or co-transfected NSP14 and NSP10 in HEK294 cells analyzed in Western blot in representative biological replicates used for RT-PCR. (**C**) RT-PCR quantifying transcript levels of NF-κB target genes *CXCL8*, *ICAM1*, *NFKBIA* and *TNFAIP3* in HEK293 cells single or co-transfected with NSP14 and NSP10. Values measured for target genes were normalized to RPII transcript levels (mean ± SD, *n*=3). (**D**) NF-κB reporter assay in HEK293 cells overexpressing IKKβ or NSP14 after treatment with 1 mM SAM for 72 h and 20 ng/ml TNFα for 18 h. Protein expression was determined by representative Western blot. (A,D) Relative NF-κB activation was determined by normalization of NF-κB-firefly luminescence to Tk-renilla luminescence (mean ± SD, *n*=3). (**E,F**) WT and D331A NSP14 expression levels in (E) HEK293 and (F) HCT116 cells with Tet-On conditional NSP14 expression system after treatment with increasing doses of DOX for 4 days. (**G,H**) NSP14 transcript levels in (G) HEK293 and (H) HCT116 cells with conditional NSP14 expression induced by 0.5 μg/ml DOX for 4 days. Values measured for NSP14 were normalized to RPII transcript levels (mean ± SD, *n*=3). (**I,J**) Stabilization of WT and D331A NSP14 in (I) HEK293 and (J) HCT116 cells after 4 days treatment with 0.5 µg/ml DOX, which induces NSP14 expression, and 6 h treatment with 25 µM MG-132. Significances were compared by (A,C,G–H) one-way ANOVA with Tukey’s multiple comparison test and (D) one-sample *t*-test after calculating log2 fold changes normalized to control treatment samples. (G,H) Asterisks mark significance of DOX treated sample compared with the untreated control sample from each cell line; *P≤0.05, **P≤0.005, ***P≤0.001, ****P≤0.0001.

To explore potential elements that enhance the NF-κB activating function of NSP14, we studied the role of its cofactors. The viral cofactor NSP10 is known to stabilize NSP14 [17] and promote its exonuclease activity [14,15]. To investigate the potential impact on NF-κB activation, we co-expressed NSP14 with NSP10 and demonstrated the stabilization of NSP14 protein by NSP10 (Figure 2A). NSP10 alone was unable to induce NF-κB activation, but NSP10 co-expression augmented the ability of NSP14 to induce NF-κB transcriptional reporter activity (Figure 2A). Next, we explored the effect of NSP14 stabilization by NSP10 on endogenous NF-κB target gene induction (Figure 2B,C). While we could not detect induction of *CXCL8* by NSP14 alone due to the low level of NSP14 expression, there was a mild induction of of *CXCL8*, *ICAM1* and *NFKBIA* transcription by NSP14 in the presence of NSP10. However, *TNFAIP3* transcript levels were unchanged and overall NSP14 stabilization by NSP10 was not able to promote a substantial induction of NF-κB target genes (Figure 2B,C). Given the importance of a functional N7-MTase catalytic center for the activation of NF-κB, we asked whether this domain could also be stabilized by a cofactor and whether this would similarly affect the regulation of NF-κB. Since the MTase domain requires the co-substrate SAM to facilitate methyl transfer, we tested whether supplementation with SAM would similarly promote NSP14 stability and functionality with respect to NF-κB. To this end, we compared the effects of SAM supplementation to the media upon overexpression of NSP14 or IKKβ, or TNFα stimulation (Figure 2D). With increased SAM supply we detected an increase in NSP14 protein and enhanced NSP14-induced NF-κB activation by approximately 2.5-fold. In contrast, TNFα stimulation similarly augmented the NSP14-induced NF-κB activation without affecting NSP14 protein levels. In IKKβ expressing cells, SAM treatment did not alter IKKβ protein amounts and only mildly increased NF-κB activation.

We consistently found that NSP14 is only expressed at very low levels for instance when compared with IKKβ, suggesting that counterbalancing mechanisms may limit NSP14 protein levels on transcriptional or post-translational level. Thus, we decided to utilize a DOX (doxycycline)-inducible expression system, which allows to study NSP14 protein dynamics. We lentivirally transduced HEK293 and HCT116 cells with NSP14 under control of the KRAB repressor that is induced by DOX incubation. DOX-treatment caused stable but low accumulation of D331A NSP14 protein, whereas WT NSP14 was barely detectable in both HEK293 and HCT116 cells (Figure 2E,F). Furthermore, low protein levels could not be enhanced by increasing the dose of DOX. We investigated whether the low NSP14 protein levels in general and the difference between NSP14 WT and D331A specifically are regulated at the transcriptional or translational level. After NSP14 WT and D331A induction with DOX, we quantified the *NSP14* transcripts by RT-PCR, which showed a strong and equivalent expression of *NSP14* WT and mutant mRNA in both cell lines (Figure 2G,H). To address, whether if the low expression of NSP14 results from post-translational instability caused by proteasomal degradation, we incubated DOX-induced HEK293 or HCT116 cells with the proteasome inhibitor MG-132. Indeed, MG-132 treatment caused an accumulation of NSP14 WT, but not the D331A mutant (Figure 2I,J). Thus, NSP14 is an inherently unstable protein and is degraded by the proteasome when overexpressed. The addition of cofactors NSP10 or SAM is able to stabilize NSP14, resulting in an accumulation and augmented induction of NF-κB.

### NSP14 is unable to activate the canonical NF-κB signaling pathway

**Figure 3:**
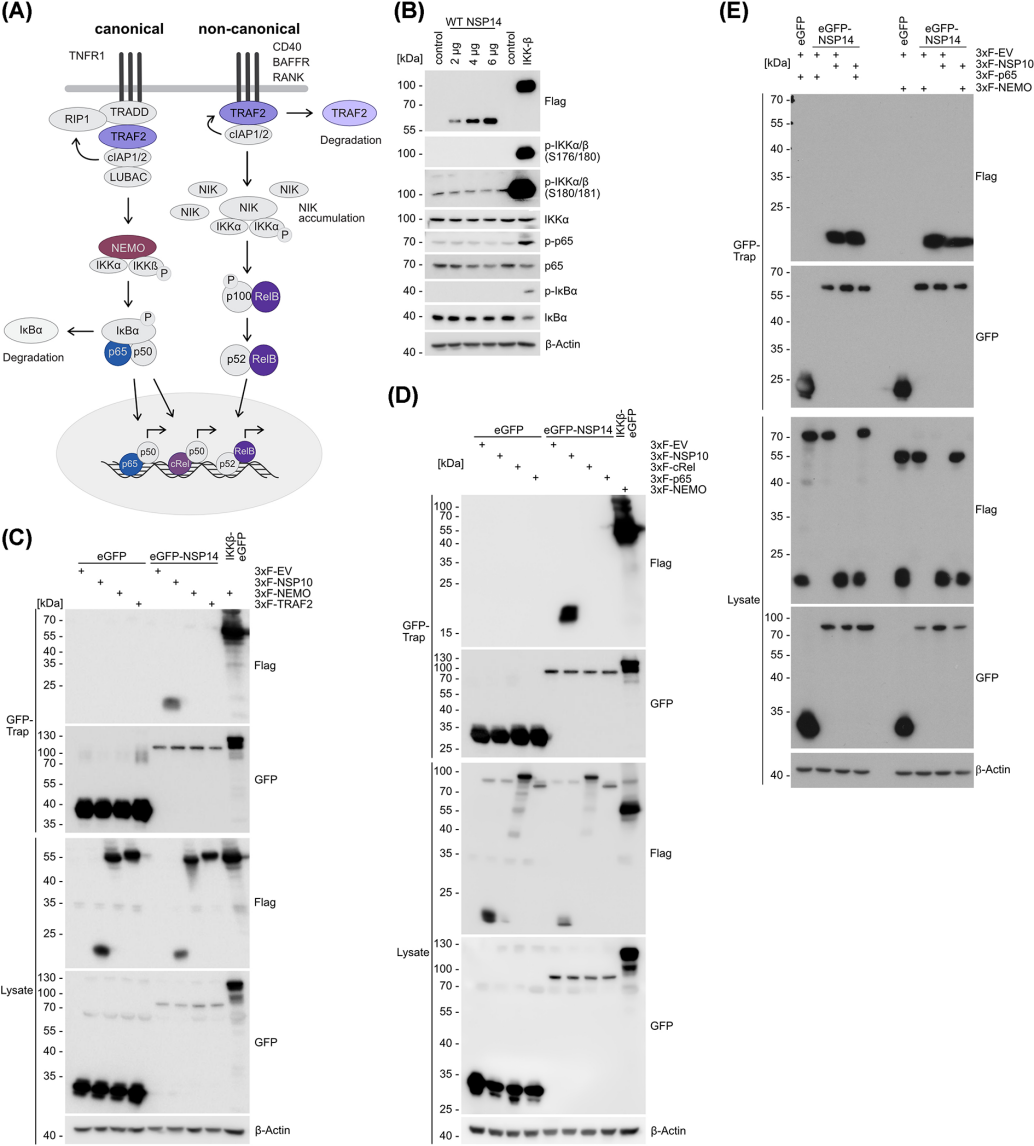
NSP14 does not stably bind putative host cell interaction partners (**A**) Scheme of the canonical and non-canonical NF-κB signaling pathways. Upon activation of the canonical pathway, upstream complexes such as TRADD, TRAF2, cIAP1/2, LUBAC and RIP1 are formed, and modification events and further protein complex formation converge in the recruitment of the IKK complex, consisting of NEMO, IKKα and IKKβ. Active IKKβ modifies IκBα, triggering its degradation, and released NF-κB dimers composed of p65, c-Rel and p50 translocate to the nucleus, initiating target gene transcription. Signals that stimulate non-canonical NF-κB signaling interfere with TRAF2 and cIAP1/2-mediated ubiquitination and degradation of NIK, inducing degradation of TRAF2 instead. Upon NIK stabilization, the alternative IKK complex of NIK and an IKKα dimer is formed, IKKα activity induces p100 cleavage, and active p52-RelB dimers translocate to the nucleus. Proteins identified in the NSP14 contactome and analyzed in this study are highlighted in color. (**B**) Analysis of canonical NF-κB signaling pathway activation (IKKα/β, p65 and IκBα phosphorylation and IκBα degradation) after overexpression of NSP14 or IKKβ using Western Blot. (**C,D**) NSP14 binding to (C) NSP10, NEMO and TRAF2 and (D) NSP10, c-Rel and p65 was analyzed after overexpression of EGFP-NSP14 with flag-tagged putative binding partners, NSP14 pull down via GFP-Traps and Western blot. (**E**) NSP14 binding to NEMO and p65 in the presence of NSP10 was analyzed as in C–D.

Systematic mapping of the SARS-CoV-2 contactome with human host cells revealed 27 human proteins that bind to NSP14 [26]. These included NEMO/IKBKG, c-Rel and TRAF2, all of which are involved in the regulation of the canonical and/or non-canonical NF-κB pathways (Figure 3A). To gained get a mechanistic understanding of how NSP14 modulates NF-κB signaling, we wanted to assess the potential of NSP14 to directly activate the canonical NF-κB signaling pathway (Figure 3B). While overexpression of IKKβ is sufficient to induce IKKα/β activation, phosphorylation of IκBα and p65 as well as degradation of IκBα protein, increasing concentrations of NSP14 were not able to activate these hallmarks of the canonical NF-κB signaling cascade. Next, we tested for interaction of NSP14 with core components of canonical NF-κB signaling, which have been identified in the NSP14 contactome map [26]. We co-expressed GFP-tagged NSP14 with 3xFLAG-tagged constructs expressing cytosolic NEMO and TRAF2 as well as nuclear c-Rel and p65 and performed GFP-TRAP pulldowns (Figure 3C,D). As expected, GFP-NSP14 associates with its viral cofactor Flag-NSP10 and GFP-IKKβ interacts with Flag-NEMO in GFP pulldowns. However, we could not detect an interaction of GFP-NSP14 and cytosolic Flag-NEMO or Flag-TRAF2 co-transfected in HEK293 cells (Figure 3C). Likewise, GFP-NSP14 did not show an association with nuclear Flag-c-Rel or Flag-p65 (Figure 3D). Since the presence of NSP10 induces conformational changes in NSP14 and enhances NSP14-driven NF-κB activation, we tested, if the presence of NSP10 may facilitate cellular interaction of NSP14 with NEMO or p65 (Figure 3E). As observed before, GFP-NSP14 interacted with Flag-NSP10, but presence of NSP10 did not augment NEMO or p65 binding to NSP14. Thus, the ability of NSP14 to activate NF-κB does not appear to depend rely on the direct stable association with previously identified interactors in the NF-κB signaling pathway.

**Figure 4:**
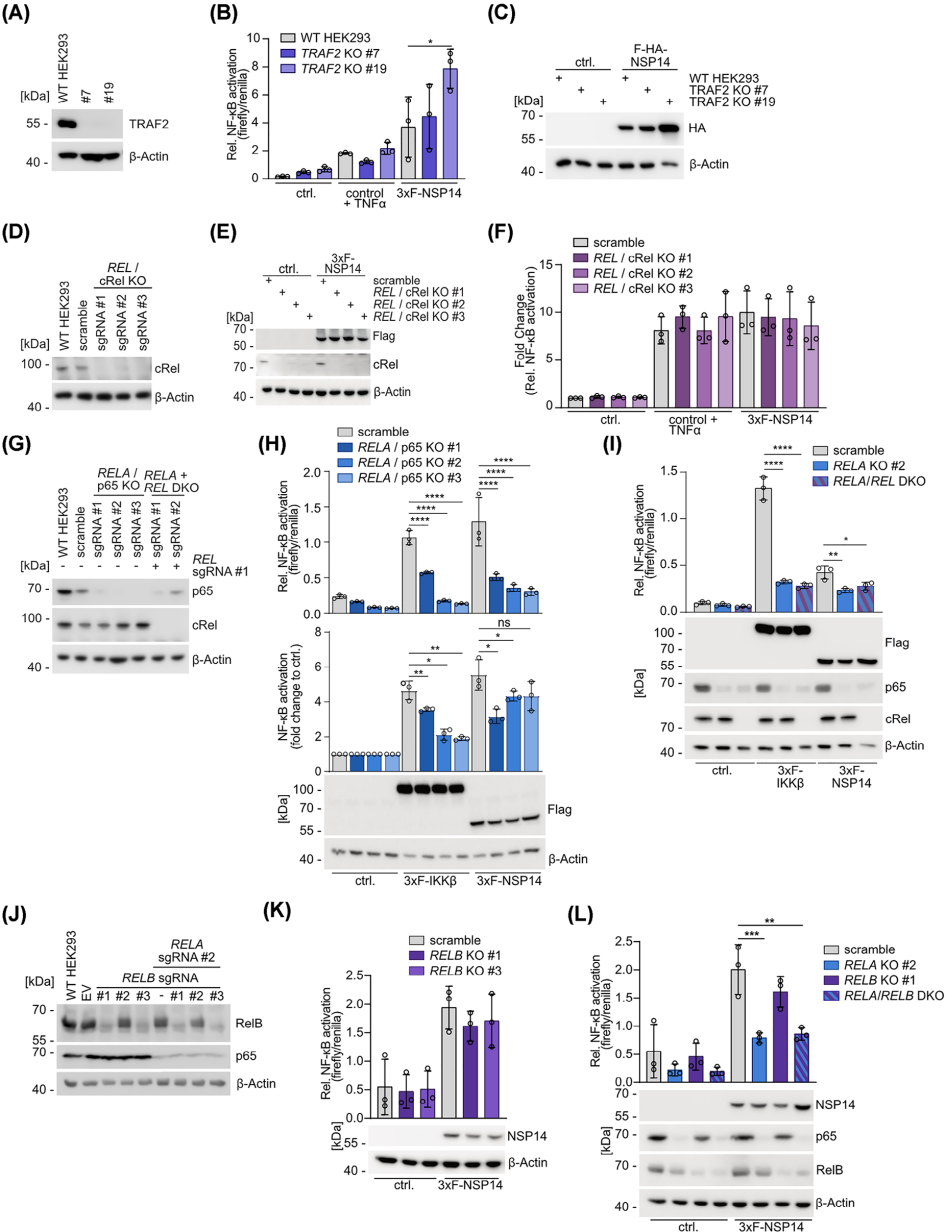
NSP14 induces canonical NF-κB activity (**A**) Detection of TRAF2 protein deficiency in *TRAF2* KO HEK293 by Western blot. (**B**) NF-κB reporter assay in *TRAF2* KO HEK293 stimulated with 20 ng/ml TNFα for 4 h or NSP14 overexpression with (**C**) representative Western blot to determine protein expression levels. (**D**) Absence of c-Rel protein in *REL* KO HEK293 cell pools compared with WT and control HEK293 cells was confirmed by Western blot. (**E**) Equal NSP14 expression levels in control and *REL* KO HEK293 cells were verified by Western blot in lysates from the NF-κB reporter assay. (**F**) Control and *REL* KO HEK293 cells were stimulated with 20 ng/ml TNFα for 4 h or NSP14 overexpression and NF-κB activity was quantified by NF-κB reporter assays. (**G**) Absence of p65 and c-Rel protein in *RELA* KO and *REL*/*RELA* DKO HEK293 compared with WT and control HEK293 was confirmed by Western blot. (**H**) NF-κB reporter assays upon overexpression of IKKβ or NSP14 in *RELA* KO HEK293. The upper panel shows relative reporter gene activity in each sample, the lower panel depicts fold changes compared with mock transfected cells. Representative Western blot to determine protein expression levels is shown. (**I**) NF-κB reporter assays upon overexpression of IKKβ or NSP14 in *REL*/*RELA* DKO HEK293 as shown in H. (**J**) RelB and p65 deficiency was tested by Western blot in *RELB* KO and *RELA*/*RELB* DKO HEK293 compared with WT and control HEK293. (**K,L**) NF-κB reporter assays in (K) *RELB* KO HEK293 and (**L**) *RELA*/*RELB* DKO HEK293 upon overexpression of NSP14 with representative Western blots to determine protein expression levels. Relative NF-κB activation is determined by normalizing NF-κB firefly luminescence to Tk renilla luminescence (mean ± SD, *n*=3). (B,F,H,I,K,L) Relative NF-κB activation is determined by normalizing NF-κB firefly luminescence to Tk renilla luminescence (mean ± SD, *n*=3). Significance was compared by two-way ANOVA followed by Tukey’s multiple comparison test (B,F,H [top] I,K,L) or by one-sample *t*-test after calculating log2 fold changes normalized to control treatment samples (H, bottom); *P≤0.05, **P≤0.005, ***P≤0.001, ****P≤0.0001.

We have previously shown that absence of NEMO, the integral non-catalytic subunit of the IKK complex which governs canonical NF-κB activation by multiple upstream pathways [33], significantly reduces, but does not completely abolish NSP14-induced activation of NF-κB [26]. Further, TRAF2 was identified as an interactor in the NSP14 contactome in yeast [26]. TRAF2 interacts with various IKK upstream receptor complexes and serves a dual role by activating the canonical NF-κB pathway (e.g. in the TNFR1 complex) or counteracting non-canonical NF-κB signaling by bridging cIAPs to degrade NF-κB inducing kinase (NIK) downstream of various receptors like CD40, BAFFR or RANK (Figure 3A) [34]. To address if absence of TRAF2 and potentially non-canonical signaling contributes to NF-κB activation by NSP14, we generated two independent *TRAF2* KO HEK293 cell clones, which we confirmed by Western blot (Figure 4A). In NF-κB reporter assays, we found slightly elevated basal levels of NF-κB in both TRAF2 KO cells, which is most likely due to its negative regulatory role in the non-canonical NF-κB pathway (Figure 4B). However, neither upon TNFα stimulation nor after overexpression of NSP14, NF-κB activation was decreased in *TRAF2* KO cells. Conversely, there was a mild enhancement of NF-κB in *TRAF2* KO clone #19, which may be due to the higher background of NF-κB activation even in the absence of stimulation, resulting most likely from its negative regulatory impact on in the non-canonical NF-κB pathway (Figure 4B,C) [34].

### NSP14-induced NF-κB activation relies on nuclear p65

In yeast-two-hybrid, NSP14 directly interacts with NF-κB family member c-Rel, which acts downstream of NEMO and forms homodimers or heterodimers (p50/c-Rel or p65/c-Rel) to bind and transactivate transcription of target genes (Figure 3A) [35]. Using the lentiCRISPRv2 system with three different *REL*-targeting sgRNAs, we generated three independent *REL* KO cell pools after puromycin-selection and found high KO efficiency as monitored by Western blot (Figure 4D). After overexpression of NSP14 in control and *REL* KO cell lines, we determined NF-κB reporter gene induction (Figure 4E,F). NF-κB activation was comparable in parental and *REL*-deficient HEK293 cells, suggesting that canonical c-Rel is not essential to mediate NF-κB activation by NSP14. Thus, we asked whether the p65 subunit, which is also able to act as a transcriptional activator of canonical NF-κB by forming nuclear homo- or heterodimers (e.g. p50/p65 or p65/c-Rel), is involved in NSP14-driven NF-κB activation either alone or in conjunction with c-Rel. We generated *RELA* KO and *REL*/*RELA* double (DKO) HEK293 cell pools using the lentiCRISPRv2 system and KO was confirmed by Western blot (Figure 4G). We first determined NF-κB activation by IKKβ and NSP14 in *RELA* single KO cells (Figure 4H). At comparable expression levels of either IKKβ or NSP14, relative NF-κB reporter gene activation by both inducers was strongly reduced in all *RELA* KO cells (Figure 4H; upper panel). Of note, we also observed a decrease in basal NF-κB reporter gene activation in *RELA* KO HEK293 cells even in the absence of IKKβ or NSP14. Strikingly, when we compared the fold change in NF-κB reporter gene activation between control (mock) and NSP14 transfected HEK293 cells, the reduction of NSP14-induced NF-κB activity in *RELA* KO HEK293 cells was mild compared with the parental cells, suggesting some residual NF-κB activity in *RELA* KO HEK293 (Figure 4H; lower panel). In contrast, both relative and fold NF-κB induction by IKKβ were strongly inhibited in *RELA* KO cells. In line with the deficiency of NSP14 to activate canonical NF-κB signaling (see Figure 3B), the data suggest that NSP14 may augment basal transcriptional activity of p65 rather than directly promoting its nuclear accumulation and DNA binding. In the RELA/REL DKO cells, we determined whether c-Rel contributes to remaining NF-κB activation in the absence of p65 (Figure 4I). However, KO of *REL* in p65-deficient cells did not further reduce NF-κB activation by either IKKβ or NSP14.

*TRAF2* KO cells indicated that non-canonical upstream signaling is not triggering NF-κB activation by NSP14, but we wanted to investigate a potential downstream contribution by also ablating RelB, the major transcriptional activator of non-canonical NF-κB signaling in p52/RelB heterodimers. Therefore, we generated *RELB* KO and *RELA/RELB* DKO cells using three different sgRNAs in the lentiCRISPRv2 system and obtained efficient *RELB* KO and *RELA/RELB* DKO HEK293 cell pools generated with sgRNA#1 and #3 as shown by Western blot (Figure 4J). Individual depletion of RelB did not affect NSP14-induced NF-κB activation (Figure 4K). Ablation of p65 strongly diminished NF-κB activation by NSP14, as previously observed, but strong decrease of RelB in *RELA/RELB* DKO cells did not further reduce NSP14-induced NF-κB activation (Figure 4L). Taken together, our data demonstrate that NSP14 drives NF-κB activity primarily by acting on the canonical p65 subunit.

## Discussion

To understand the molecular mechanism by which NSP14 activates host cell NF-κB signaling, we have systematically evaluated the domains and enzymatic functions of NSP14. Partial or complete truncation of either domain fully abolished NF-κB activation. In contrast to the mutation of the ExoN catalytic center, genetic inactivation of the MTase catalytic center completely inhibited NSP14-induced NF-κB activation. Thus, the MTase catalytic activity of NSP14, which requires full-length NSP14 for full functionality [19], is necessary for the activation of NF-κB.

Beyond the importance of the MTase domain activity, we demonstrate that cofactors of both NSP14 domains, SAM and NSP10, increase the NSP14 protein levels. This aligns with other studies reporting the stabilization of NSP14 upon co-expression of NSP10 [16,17] and addition of S-Adenosylhomocysteine (SAH), which is the product of the MTase reaction [17]. So far, there is no structural analysis of NSP14 in complex with SAH but we speculate that the stabilization may be facilitated by strengthening the conformation of the flexible SAM binding region [11]. The *in vitro* comparison of SARS-CoV-2 proteins has previously shown that NSP14 activates NF-κB in a dose-dependent manner [26]. Similarly, the viral load detected in patients correlates with the degree of their inflammatory response [6,29]. Hence, we speculate that the augmented NF-κB activity is based on the accumulation of NSP14 protein, although the increase in co-substrate may also increase the enzymatic activity of MTase. However, SAM supplementation and thus the promotion of MTase activity, generally affects the cellular SAM/SAH homeostasis, which can also modulate NF-κB transcriptional activity independently from NSP14 [36]. Using the inducible NSP14-expressing cells, we found that NSP14 is unstable and degraded by the proteasome. In particular, the active NSP14 protein is highly unstable, suggesting that host cells tightly control the expression, stability and activity of NSP14 as it may negatively affect cellular functions. Binding of the viral factor NSP10 stabilizes NSP14 and increases its potential to activate NF-κB, arguing for a high cooperativity between viral factors to modulate human immune and inflammatory responses.

In addition to the central role of NSP14 methyltransferase activity, we show that p65 is essential for NSP14-induced NF-κB activity, whereas it is not dependent on the NSP14-binding factor c-Rel. This is consistent with other studies showing activation of p65 upon *in vitro* SARS-CoV-2 infection [31] and expression of NSP14 [27]. However, even though NEMO is critical for NSP14-triggered NF-κB activation, NSP14 overexpression is unable to induce upstream canonical IKK/NF-κB signaling. In fact, our data indicate that NSP14 enhances basal NF-κB transcriptional activation primarily by acting on p65. In line, *IKBKG*/*NEMO* KO severely reduces constitutive NF-κB activation and thus also leads to a diminished NSP14 response [26]. Even though we did not see a contribution of c-Rel or RelB in *REL*/*RELA* or *RELB*/*RELA* DKO cells, other family members such as p50 or p52 or a combination of multiple NF-κB factors must contribute as suggested by the residual NSP14-induced NF-κB activation in the absence of p65. Interestingly, *in vitro* experiments in A549 lung epithelial cells have shown that both c-Rel [26] and p65 [31] are required for SARS-CoV-2 replication, indicating that NF-κB activation upon infection may not only drive the cytokine storm but is also essential for propagation of SARS-CoV-2. It remains to be investigated whether NSP14-triggered NF-κB activation is responsible for driving SARS-CoV-2 replication and whether cell type-specific differences may account for variations in the requirement for c-Rel and p65. In sum, NSP14 activates constitutive canonical NF-κB transcriptional activity, which involves primarily NEMO and nuclear p65 [26].

Furthermore, we find that augmentation of canonical NF-κB by NSP14 only minimally affects transcription of endogenous NF-κB target genes, as reflected by only weak activation of *CXCL8*, which was previously shown by Zaffagni et al. [28]. This is in contrast to *in vitro* SARS-CoV-2 infection of A549 cells, which promotes transcription of numerous NF-κB target genes [31]. We speculate that this difference is related to the fact that our firefly reporter plasmid is transiently expressed outside the genomic surrounding and thus may be directly accessible, whereas NF-κB binding sites in the promoter regions of endogenous loci are inaccessible in the chromatin. Thus, NSP14 alone is capable of inducing NF-κB activity, but additional viral or host factors are required for full induction of target genes, which may include pathways for opening nuclear chromatin. In our model, the NSP14-induced NF-κB activation may lower the threshold for full activation of NF-κB. In combination with other SARS-CoV-2-induced inflammatory signals, this may promote mild activation of NF-κB and initiate chemokine and cytokine expression. With positive feedback loops, a secondary, stronger activation of NF-κB and release of more cytokines may be triggered in a vicious cycle of inflammation.

There are some limitations to our study. For technical reasons, we are only able to study the effect of NSP14 on NF-κB activation outside the context of a full viral infection. Upon overexpression, cellular concentration, localization and activity of NSP14 may differ considerably from that of a SARS-CoV-2 infected human cell and may not fully recapitulate the processes after viral transduction. However, studies utilizing overexpression provided important insights how individual viral factors contribute to SARS-CoV-2 pathogenicity and how this may be exploited therapeutically (see [20]). Further, we have investigated basic mechanisms of NSP14-triggered NF-κB activation in HEK293 cells, which as a human kidney cell line is not a primary host cell of SARS-CoV-2. However, HEK293 cells contain all core NF-κB components and the cells were chosen to best compare our data with previous studies that also utilized HEK293 cells [26–28]. We cannot exclude cell-type specific differences, but previous data provided evidence that NSP14 also activates NF-κB in A549 lung epithelial cells [27]. In the future, it will be important to dissect mechanisms of NF-κB activation in cells that are primarily infected by SARS-CoV-2, such as pharyngeal or bronchial epithelial cells. Finally, we have not been able to elucidate the exact mechanism(s) how NSP14 induces NF-κB transcriptional activation via p65. The necessity of NSP14 MTase activity and the lack of detectable stable interactions with NF-κB regulators upon overexpression in HEK293 cells suggests that NSP14 methyltransferase activity may induce NF-κB activation indirectly by capping viral or potentially human host mRNAs, but the direct substrates in this process remain to be identified. It has been shown previously that coronaviral NSP14 does not require a characteristic viral RNA sequence or structure for target recognition and is indeed able to substitute for homologous enzymes in other organisms [37]. Of particular interest will be which mRNAs are affected, whether NSP14 acts in a selective or non-selective manner with respect to NF-κB activation, and how this leads to NF-κB induction.

Taken together, we show that the enzymatic activity of NSP14 MTase is critical for NF-κB activation, which enhances basal NF-κB activation through NEMO/IKK complex and p65. Inhibitors targeting the NSP14 MTase domain have been proposed based on *in vitro* screens, but most compounds have not been further tested for specificity against SARS-CoV-2 MTases [38–41]. Development of effective inhibitors that selectively interfere with NSP14 MTase activity may be of therapeutic interest for treating COVID-19 patients and may also help to better understand the regulation and function of NF-κB activation by NSP14.

## Methods

### Cell lines and growth conditions

Human embryonic kidney (HEK) 293T (RRID: CVCL_0063, DSMZ), HEK293 (RRID: CVCL_0045, DSMZ), and HCT116 (RRID: CVCL_0291) cells were maintained at 37°C and 5% CO_2_ in a humidified atmosphere. All cell lines were cultured in Dulbecco’s modified Eagle’s medium supplemented with 10% fetal bovine serum (FBS) and 100 U/ml penicillin/streptomycin and passaged at 80% confluence. After trypsinization with 0.05% trypsin/EDTA, cells were seeded at appropriate densities for experiments.

### Generation of DNA constructs

NSP14 and NSP10 cDNA sequences were amplified and mutations were introduced by site-directed mutagenesis using WT, δ- and ο-variant NSP14 and WT NSP10 constructs (based on SARS-CoV-2 Wuhan-Hu-1, NCBI reference NC_045512.2). Expression vectors for protein overexpression were cloned by inserting NSP14, NSP10, REL (Addgene #27253) [42] and RELA (Addgene #21966) [43] cDNAs into a modified pEF4 (Thermo Scientific) or pEGFP-C1 (Clontech) using conventional restriction enzyme cloning. Amplified WT and D331A NSP14 were cloned into pLVTHM (Addgene #12247) [44] using NEBuilder cloning. Primer sequences used for the cloning steps are summarized in Table 1. Lentiviral transfer vectors lentiCRISPRv2 (Addgene #52961) [45] containing sgRNAs targeting REL, RELA or RELB and pSpCas9(BB)-2A-GFP (PX458) (Addgene #48138) [46] containing sgRNA targeting TRAF2 were cloned by common restriction enzyme cloning. 3xF-IKKβ in pRK5, 3xF-NEMO in modified pcDNA3.1, IKKβ in pEGFP-C1 and 3xF-TRAF2 in pEF4 were described elsewhere [47]. The plasmids were amplified in TOP10 or Stbl3 chemically competent *E. coli.*

**Table 1:** Summary of oligonucleotides used for cloning

Name	Sequence (5′->3′)
NSP14 fw	CTAGTGGATCCGCTGAAAATGTAACAGGAC
NSP14 rv	ACTAGGCGGCCGCTTACTGAAGTCTTGTAAAAG
NSP14 ΔN fw	CTAGTGGATCCGAAGAAGCTATAAGACATG
NSP14 ΔExoN fw	CTAGTGGATCCAAGCGTGTTGACTGGAC
NSP14 ΔMTase rv	ACTAGGCGGCCGCTTAAACAAAGCACTCGTGGAC
NSP14 D90A E92A rv	GACACCCCGCGACAGCGAAGCC
NSP14 D331A fw	GTTCTTCACGCCATTGGTAAC
NSP10 fw	TACTGTCTAGAGCTGGTAACGCTACTGAGGTG
NSP10 rv	TACTGTGTCGACTTACTGCAGCATAGGTTCCCTCAG
REL fw	TACTGGGATCCGCCTCCGGTGCGTATAACCCGTATATAGAGATCATCGAGCAG CCTAGACAAAGAGGAATGCGTTTTAGATACAAATGTGAAGGG
REL rv	CAGTAAGCGGCCGCTTATACTTGAAAAAATTCATATGG
RELA fw	TACTGGGATCCGACGAACTGTTCCCCCTC
RELA rv	CAGTAAGCGGCCGCTTAGGAGCTGATCTGACTCAG
EGFP-NSP14 fw	ACTGATCTCGAGCTGCTGAAAATGTAACAGGACTC
EGFP-NSP14 rv	TATCGGGATCCTTACTGAAGTCTTGTAAAAGTGTTCC
Tet NSP14 fw	ACGAGACTAGCCTCGAGGTTTAAACGCCACCATGGCTG AAAATGTAACAGGACTC
Tet NSP14 rv	CCTTCACAAAGATCCTCATATGTTACTGAAGTCTTGTAAAAGTGTTCC

### Generation of inducible cell lines

To generate DOX-inducible NSP14-expressing HEK293 and HCT116 cells, two sequential viral infections were performed: First, the TetR-KRAB cassette was integrated (pLV-tTRKRAB-red), while the tetracycline response element-controlled NSP14 expression constructs (pLVTHM- empty (control), pLVTHM-WT NSP14, pLVTHM-D331A NSP14) were integrated in the second step. For lentivirus production, 450,000 HEK293T cells per 6-well were seeded in 4 ml media and grown overnight. The next day, cell transfection was performed using 0.25 μg psPAX2 (Addgene #12260), 0.17 μg pMD2.G (Addgene #12259), 0.35 μg plasmid of interest with X-tremeGENE HP DNA Transfection Reagent (Roche, Cat# 06366244001) according to the manufacturer’s protocol. After three days of virus production, virus-containing supernatant was filtered (0.45 μm). Viral transduction was performed by adding 2 ml virus-containing supernatant and 2 μl Polybrene (Merck, TR-1003-G) to 480,000 HEK293 or HCT116 cells per 6-well. Following three days of incubation, the transduced cells were washed twice with PBS. Successful infection and generation of >90% HEK293 KRAB and HCT116 KRAB was confirmed by fluorescence microscopy. After the second infection, the freshly washed cells were treated with 1.0 μg/ml DOX (Sigma Aldrich, Cat#D9891) for 3 days and selected with puromycin (Sigma Aldrich, Cat#540411) by addition of 1.0 μg/ml DOX + 1.0 μg/ml puromycin for 48 h. Afterward, cells were maintained under standard conditions and NSP14 expression was induced individually by the addition of 0.5 μg/ml DOX.

### Generation of KO cell lines

*REL*, *RELA*, *RELB* KO HEK293 cells were generated using the lentiCRISPRv2 system. Targeting oligos (*RELA*: 5′ CAAGTGCGAGGGCGCTCCG 3′, 5′ TATCTGTGCTCCTCGCCT 3′ and 5′ TCACCAAGGACCCTCCTCAC 3′; *RELB*: 5′ AACGGCTTCGGCCTGGACGG 3′, 5′ CTCCTCACTCTCGCTCGCCG 3′ and 5′ GCCACGCCTGGTGTCTCGCG 3′) were inserted into the lentiCRISPRv2 backbone, for control cell lines (‘scramble’) an empty backbone without sgRNA was ligated. LentiCRISPRv2 constructs targeting *REL* were containing the following sgRNAs: *REL*: 5′ ATTGGGTTCGAGACAACAGG 3′, 5′ TAATTGAACAACCCAGGCAG 3′ and 5′ GTTGGAAAAGACTGCAGAGA 3′. Lentivirus production and cell transduction were performed as described above using 2 × 10^6^ HEK293T cells in 9 ml media per 10 cm dish. Cell transfection was performed with 1.5 μg psPAX2, 1.0 μg pMD2.G, 2.0 μg lentiCRISPRv2. Viral transduction was performed by adding 1 ml (*REL* KO) or 3 ml (*RELA*, *RELAB* KO) virus and 1 or 3 μl Polybrene to 350,000 HEK293 cells per 6-well. For DKO infections, virus and polybrene volume were doubled. Transduced cells were selected with 1.0 μg/ml puromycin for 48 h and maintained with 1.0 μg/ml puromycin. *TRAF2* KO HEK293 cells were generated as single cell clones as previously described [26] using sgRNAs 5′ GCCGGGCTGTAGCAACTCCA 3′ and 5′ AGGCCCTTCCAGGCGCAGTG 3′. Successful KO was verified by analyzing protein expression by Western blot.

### NF-κB reporter assays

Cell transfection and NF-κB reporter assays have been described in detail [26]. Briefly, one day after seeding, cells were transfected with a calcium phosphate protocol using 10 ng NF-κB firefly luciferase reporter plasmid [48], 50 ng pRL-TKluc reporter plasmid (Promega), and expression vectors in a total of 6 μg DNA (supplemented with empty vector). Twenty-four hours after transfection or after treatment as indicated, NF-κB activity was quantified using the dual luciferase reporter kit (Promega, E1980) according to the manufacturer’s protocol. Luminescence was measured with a luminometer (Berthold Centro LB960 microplate reader, MikroWin 2010 software), and data analysis was performed in GraphPad Prism v7.04.

### Cell stimulation and treatment

For SAM supplementation, 1 mM S-adenosylmethionine (SAM) (Sigma Aldrich, A4377) was added when the media was changed after transfection. SAM treatment was incubated for 72 h, and 20 ng/ml TNFα (Biomol, Cat# 50435) was added for the last 18 h before lysis. MG-132 (Merck, Cat#474790) treatment was performed for 6 h at a final concentration of 25 μM. TNFα stimulation was performed with 20 ng/ml TNFα for 4 h starting 24 h after transfection.

### Protein binding studies

For protein binding studies, 2.5 × 10^6^ HEK293 cells were plated in 10 cm dishes and transfected the next day with 7.5 μg pEGFP-C1 and 5 μg 3xFlag-pEF4 constructs with a calcium phosphate protocol. After 24 h incubation, lysis and GFP-Trap (Chromotek, Cat#gta) were performed as described [49]. Different to the referred approach, the beads were washed six times in Co-IP buffer after incubation with the lysate.

### Cell lysis

For analysis of protein levels, cells were washed once with ice-cold PBS. Lysis was performed with 100 μl of high salt buffer (20 mM HEPES pH 7.9, 350 mM NaCl, 20% glycerol, 1 mM MgCl2, 0.5 mM EDTA, 0.1 mM EGTA, 1% NP-40, 1 mM DTT, 10 mM sodium fluoride, 8 mM β-glycerophosphate, 300 μM sodium vanadate, and Roche protease inhibitor cocktail [Roche, Cat#11836145001]) per confluent 6-well. Protein expression in NF-κB reporter assays and GFP-Traps was analyzed in the lysates of the respective assays. All lysates were denatured with 4xSDS loading dye and boiled.

### Western blotting

Proteins were separated by SDS-PAGE and transferred to PVDF membranes. After blocking in 5% milk in 1 × PBS + 0.1% Tween-20 (PBS-T) for 1 h at room temperature (RT), membranes were incubated with primary antibodies (indicated below, in 2.5% BSA in PBS-T) overnight at 4°C. The next day, membranes were washed three times in PBS-T, and secondary antibodies (indicated below, in 1.25% milk in PBS-T) were incubated for 1 h at RT. Detection was performed using HRP-catalyzed enhanced chemiluminescence and LumiGlo reagent (CST, 7003S), data were collected with an ECL Chemocam Imager (INTAS) and the ChemoStar Software (INTAS). Full uncropped versions of Western blots are shown in Supplementary Figure S1.

The following primary antibodies were used in a dilution of 1:1000 when not stated differently: Flag (M2) (Cat#F3165; RRID: AB_259529), HA (Cat#11583816001, RRID:AB_514505) from Sigma Aldrich; β-Actin (C4) (Cat#sc‐47778; RRID: AB_626632; 1:5000), c-Rel (Cat#sc-70, RRID:AB_2178727), p65 (C-20) (Cat#sc‐372; RRID: AB_632037) from Santa Cruz Biotechnology; NSP14 (Cat#99098), GFP (Cat#2555, AB_221569), TRAF2 (Cat#4712, RRID:AB_2209848), RelB (Cat#4922, RRID:AB_2179173), p-IKKα/β (S176/180) (Cat#2697; RRID: AB_2079382), p-IKKα/β (S180/S181) (Cat#2681; RRID: AB_331624), IκBα (L35A5) (Cat#4814, RRID: AB_390781), p-IκBα (S32/36) (5A5) (Cat#9246; RRID: AB_2151442), p-p65 (S536) (Cat#3033; RRID: AB_331284) from Cell Signaling Technology; IKKα (14A231) (Cat#05-536; RRID: AB_11213043) from Merck Millipore. The following secondary antibodies were used in a dilution of 1:10,000: HRP‐conjugated anti‐rabbit (Cat#715‐035‐150; RRID: AB_2340770), HRP‐conjugated anti‐mouse (Cat#112‐035‐062; RRID: AB_2338133) from Jackson ImmunoResearch.

### Quantitative reverse-transcriptase polymerase chain reaction (qRT-PCR)

For gene expression analysis, HEK293 cells were transfected with empty vector, NSP14 or IKKβ or gene expression was induced by addition of DOX as described above. Transfected cells were harvested as follows: after addition of 5 volumes of Trizol (Invitrogen, Cat# 15596018) and incubation for 5 min (min) at RT, 1 volume of chloroform was added and the sample was shaken vigorously. After 3 min incubation, samples were centrifuged (5 min, 20,000 × ***g***, 4°C) and the colorless aqueous phase was used for RNA isolation. Samples were homogenized (QIAGEN QIAshredder spin columns, Cat#79656) and RNA was isolated (QIAGEN RNeasy Kit, Cat# 74004). After DOX-induced gene expression, RNA was harvested using the QIAGEN RNeasy Kit. cDNA was transcribed using the Verso cDNA synthesis kit (AB1453B, Thermo Fisher Scientific) and equal amounts of RNA. All kits were used according to the manufacturer's protocol. Quantitative real-time PCR was performed using KAPA SYBR FAST qPCR Master Mix (KAPA Biosystems, Cat# KK4600) on a Roche LightCycler 480. A standard LightCycler protocol was implemented and RNA Polymerase II (RPII) was used as an internal standard. The following primers and annealing temperatures were used: RPII fw: 5′-GCACCACGTCCAATGACA-3′ rev: 5′-GTGCGGCTGCTTCCATAA-3′, Tm 64°C; CXCL8 fw: 5′CTTGGCAGCCTTCCTGATTT 3′, rev: 5′GGGTGGAAAGGTTGGAGTATG 3′ (Li 2022), Tm 60°C; ICAM: fw: 5′GGCTGGAGCTGTTGAGAAC 3′, rev: 5′ACTGTGGGTTCAACCTCTG 3′, Tm 64°C; NFKBIA: fw: 5′ AGGACGGGGACTCGTTCCTG 3′, rev: 5′ CAAGTGGAGTGGAGTCTGCTG 3′, *T*m 64°C; TNFAIP3: fw: 5′ CTGAAAACGAACGGTGACGG3′, rev: 5′CGTGTGTCTGTTCCTTGAGCG3′, *T*m 64°C; NSP14 fw: 5′ CGGAAACCCAAAGGCTATCA 3′, rev 5′ TGTGGGTAGCGTAAGAGTAGAA 3′, *T*m 60°C.

### Statistical analysis

NF-κB reporter assays and RT-PCR were performed with a sample size of *n*=3, and data are presented as mean ± standard deviation (SD). To compare two groups, statistical significance was calculated using the unpaired Student’s *t*-test (Figure 1F). Data with more than two groups and one variable were analyzed by one-way analysis of variance (ANOVA) combined with Tukey’s (Figures 1B,C,G and 2A,C,G,H) or Dunnett’s (Figure 1D) multiple comparison test. Two-way ANOVA with Tukey’s multiple comparison test was used for data with more than two groups and two variables (Figure 4B,F,H [top], I,K–L). Data shown in Figures 2D and 4H (bottom) were normalized to control treatment samples, and statistical significance was compared by calculating log2 fold changes to normalizations and one-sample *t*-test. *P-*values indicating statistical significance are indicated by asterisks: *P≤0.05, **P≤0.005, ***P≤0.001, ****P≤0.0001. Data analysis was performed in GraphPad Prism v7.04.

## Supplementary Material

Supplementary Figure S1

## Data Availability

All materials, data and associated protocols will be made available upon request.

## Abbreviations

COVID-19: coronavirus disease 2019
CoV: coronavirus
DOX: doxycycline
ExoN: exonuclease
GFP: green fluorescence protein
IKK: Inhibitor of κB kinase
KO: knockout
MERS: middle east respiratory syndrome
MTase: methyltransferase
NEMO: NF-κB essential modulator
NF-κB: nuclear factor κB
NSP: non-structural protein
ORF: open reading frame
qRT-PCR: quantitative reverse transcriptase polymerase chain reaction
RTC: replication-transcription complex
SAM: S-adenosylmethionine
SARS: severe acute respiratory syndrome
TNF: tumor necrosis factor
TRAF: TNF receptor associated factor
