# Supplementary material for: SARS-CoV-2 NSP14 MTase activity is critical for inducing canonical NF-κB activation
Source: Biosci Rep. 2024 Jan 9;44(1):BSR20231418. doi: 10.1042/BSR20231418 (PMC10776897; doi:10.1042/BSR20231418)
Supplement: Supplementary Figure S1 [file BSR-2023-1418_supp.pdf]

Figure 1B

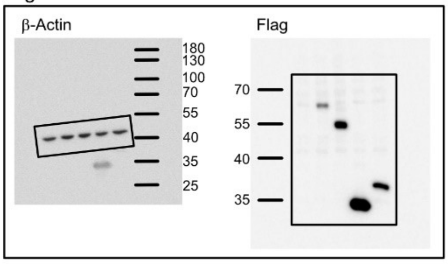

Figure 1C

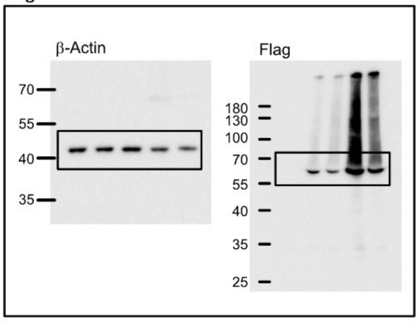

Figure 1D

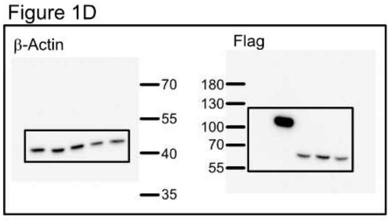

Figure 1E

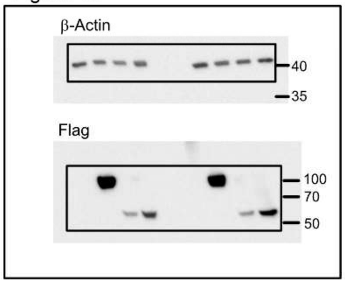

Continued next page

Figure 2A

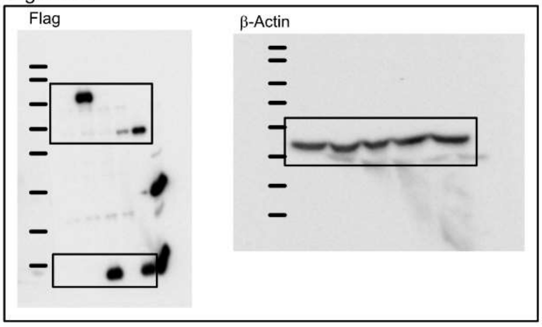

Figure 2B

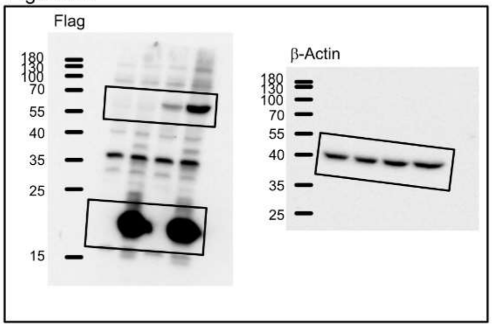

Figure 2D

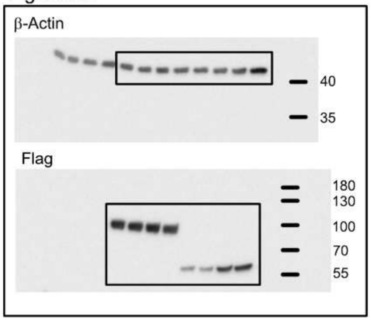

Figure 2E

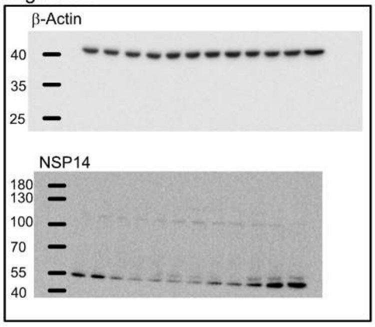

Figure 2F

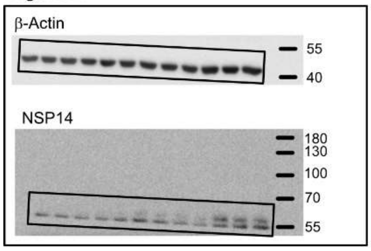

Figure 2I

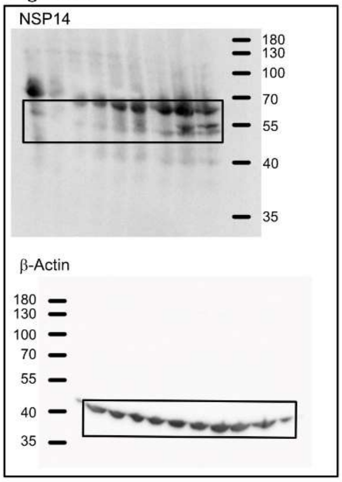

Figure 2J

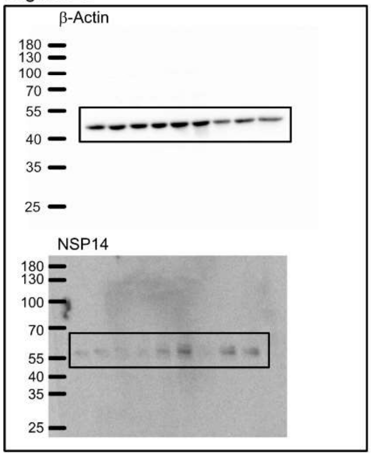

Continued next page

Figure 3B

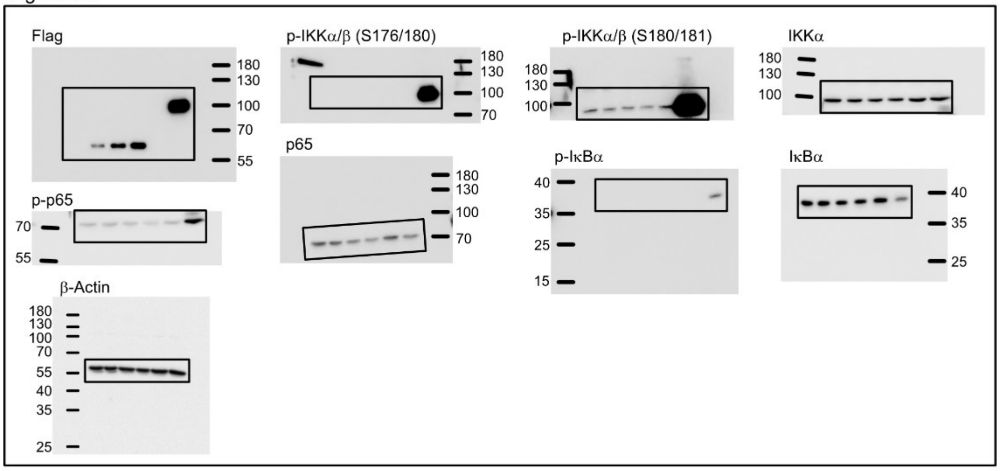

Figure 3C

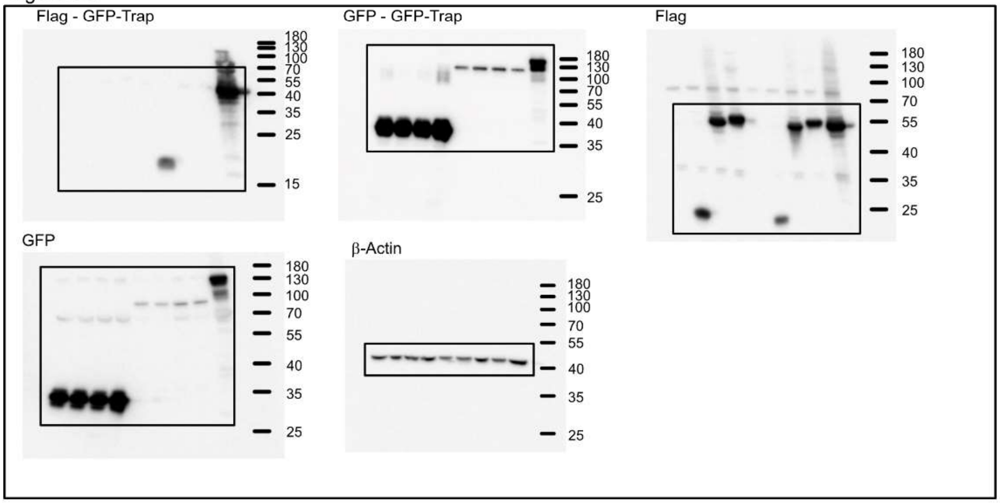

Figure 3D

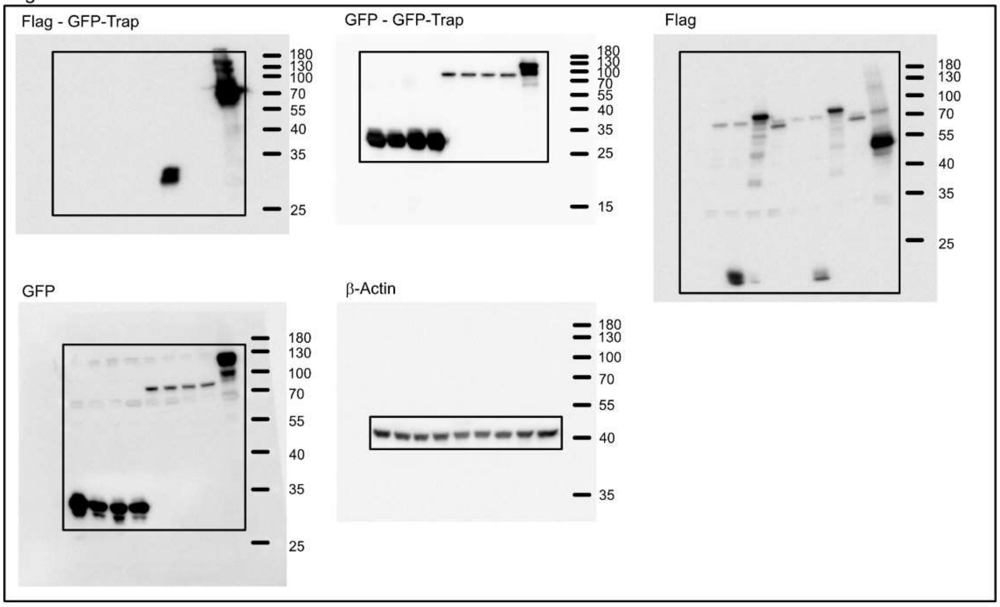

Continued next page

Figure 3E

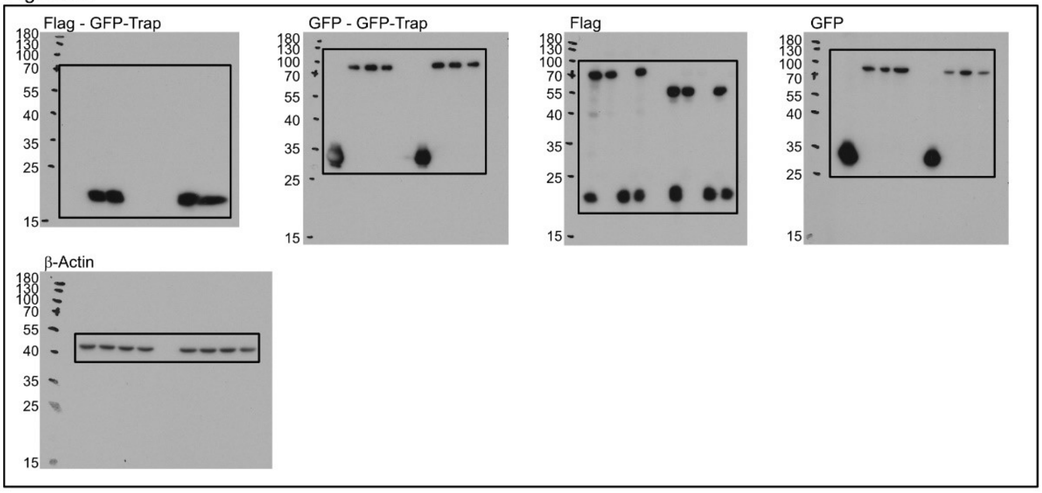

Continued next page

Figure 4B

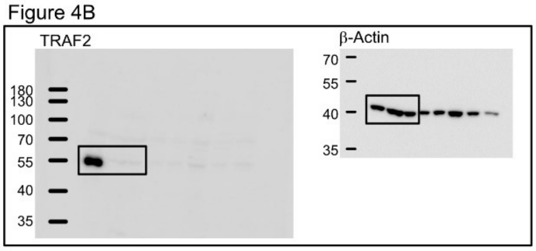

Figure 4C

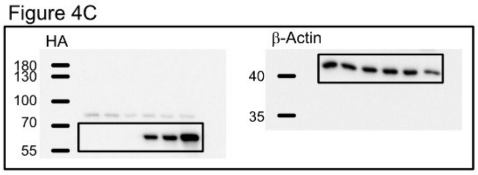

Figure 4D

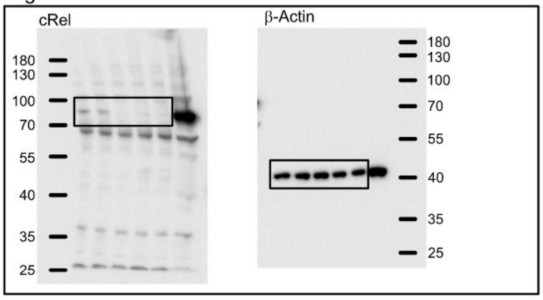

Figure 4E

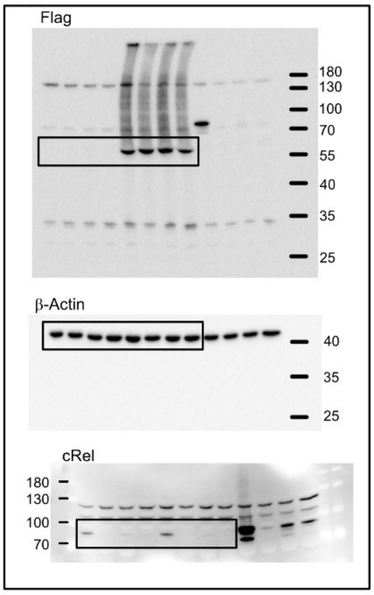

Figure 4G

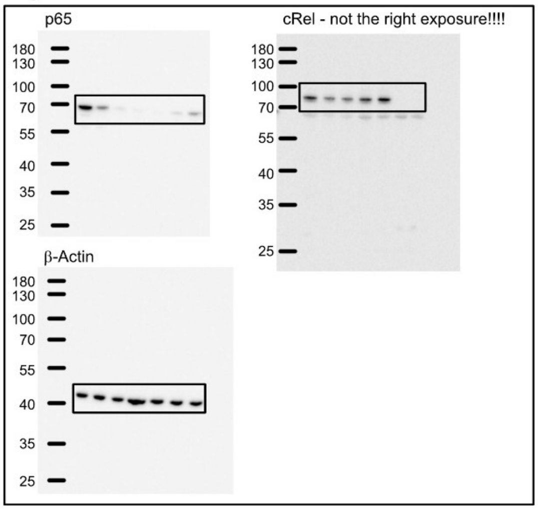

Figure 4H

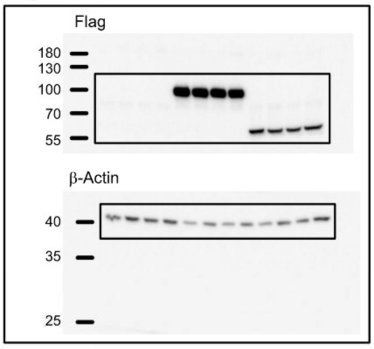

Figure 4I

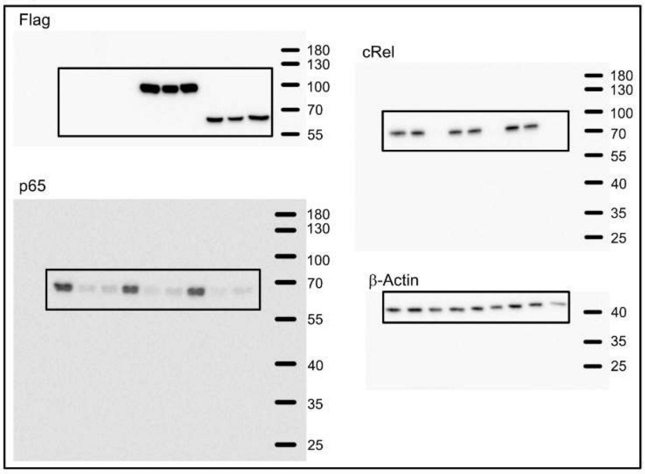

Figure 4J

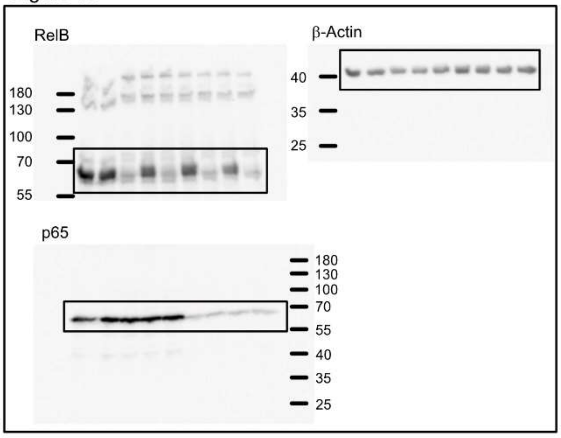

Continued next page

Figure 4K

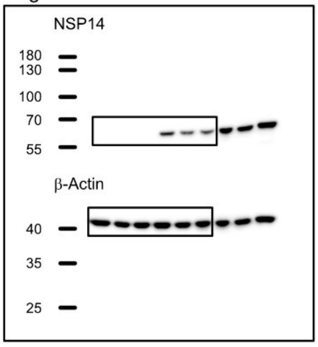

Figure 4L

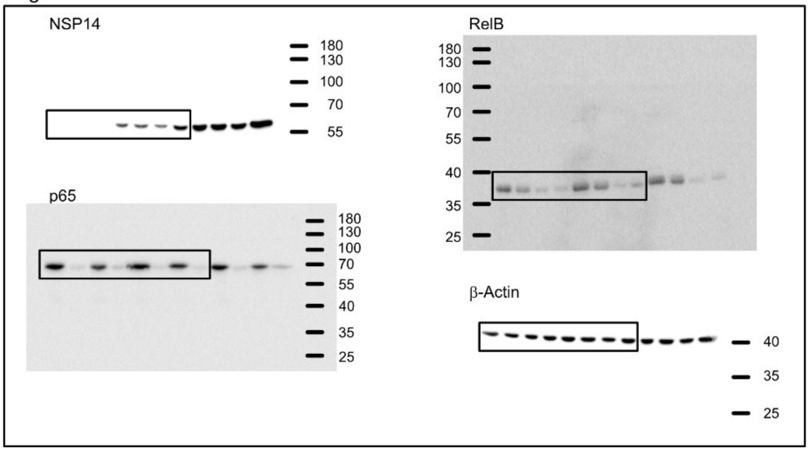

Supplementary Figure 1: Full uncropped versions of Western blots. Western blot images were collected with an ECL Chemocam Imager (INTAS) and analyzed with ChemoStar Software (INTAS). Molecular mass markers are not directly visualized in the ECL imager and have been marked subsequently in the images.
